# Transcription Factor SsNdt80b Maintains Optimal Expression of *SsSNF1* to Modulate Growth and Pathogenicity in *Sclerotinia sclerotiorum*

**DOI:** 10.1111/mpp.70088

**Published:** 2025-04-19

**Authors:** Wenli Jiao, Tianyi Lei, Qingyu Duan, Jingyuan Wang, Yushan Yang, Guang Li, Rongbao Zhang, Hongyu Pan, Yanhua Zhang

**Affiliations:** ^1^ College of Plant Sciences Jilin University Changchun China; ^2^ Baicheng Academy of Agricultural Sciences Baicheng China; ^3^ Jilin Province Bada Pesticide Co. Ltd. Gongzhuling China

**Keywords:** cell wall–degrading enzymes (CWDEs), Ndt80, pathogenicity, *Sclerotinia sclerotiorum*, SsSnf1

## Abstract

Microorganisms use versatile strategies to facilitate the colonisation of hosts, through remodelling transcription and metabolism to accommodate growth under harsh and hostile environments. *Sclerotinia sclerotiorum* is a typical necrotrophic pathogen that causes Sclerotinia stem rot in more than 700 species, resulting in serious economic losses. How *S. sclerotiorum* integrates mechanisms for nutrient acquisition and utilisation to maintain optimal growth and pathogenicity is still indistinct. Here, we demonstrate that Ndt80 family transcription factors (SsNdt80a,b,c) are involved in carbon source utilisation and have different roles in the growth, sclerotia formation, infection cushion development, and the virulence of *S. sclerotiorum*. SsNdt80b could bind the promoter of *SsSNF1* and modulate the transcriptional activity of *SsSNF1.* Silencing *SsSNF1* resulted in defects in hyphal growth and infection cushion formation, reduced cell wall‐degrading enzymes, and reduced pathogenicity of *S. sclerotiorum*. A model is proposed in which SsNdt80b responds to carbon sources and modulates SsSnf1 to regulate the development and pathogenicity of *S. sclerotiorum*.

## Graphical abstract

This study reveals that SsNdt80b regulates carbon utilisation and pathogenicity in *Sclerotinia sclerotiorum* by modulating *SsSNF1*, affecting growth, development and virulence.

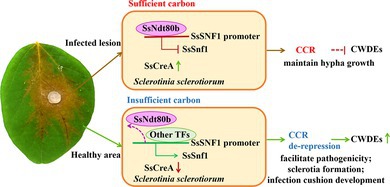

## 1 Introduction

*Sclerotinia sclerotiorum*, a necrotrophic plant pathogen, penetrates plant tissue via an infection cushion and uses dead plant biomass as a nutrient source to support rapid growth (Derbyshire et al. 2022; Shang et al. 2024). *S. sclerotiorum* can infect more than 700 plant species and is responsible for economic losses of crops, including rapeseed, soybean and sunflower. Because of the lack of cultivar resources with high resistance to *S. sclerotiorum*, managing Sclerotinia disease is still challenging. Thus, understanding the genetic mechanisms that regulate how *S. sclerotiorum* responds to the environment and assimilates nutrients is important for identifying effective strategies to control disease.

Ndt80/PhoG‐like transcription factors (TFs) had been reported to be involved in the regulation of meiosis, virulence, response to nutrient stress and sexual development (Doyle et al. 2016; Katz 2019; Shahi et al. 2016; Xie et al. 2016). However, Ndt80 TFs in different fungi are highly variable (Nocedal et al. 2017). In *Saccharomyces cerevisiae* , Ndt80 (Nuclear division transcription) is triggered by nutrient limitation and required for activating gene transcription during meiosis (Winter 2012). CaNdt80 is required for antifungal drug resistance, hyphal growth and virulence of *Candida albicans* (Chen et al. 2004). In *Neurospora crassa*, *VIB1* represses both glucose signalling and carbon catabolite repression (CCR) under carbon‐limited conditions, thus enabling a proper cellular response for plant biomass deconstruction and utilisation (Xiong et al. 2014). Because there are significant differences in the composition and function of Ndt80 family TFs among different species (Doyle et al. 2016; Shahi et al. 2016; Yang et al. 2022), clarifying the function of Ndt80 family TFs in *S. sclerotiorum* is conducive to a more comprehensive understanding of the role of the Ndt80 family during the pathogenic mechanism of plant pathogens.

CCR, a global transcriptional regulatory system of most microorganisms, represses carbon source utilisation when a dominant carbon source (such as glucose) is present and controls the synthesis of enzymes (Wu et al. 2020). Whether CCR influences the growth and pathogenicity of *S. sclerotiorum* is currently unknown. CCR operates via a regulator to ensure glucose is preferentially utilised (Dowzer and Kelly 1991; Flipphi et al. 2003; Mathieu and Felenbok 1994), Mig1p is a core regulator in glucose repression, recruiting general repressors Ssn6p and Tup1p and binding with the promoters of glucose‐repressed genes in *S. cerevisiae* (Ronne 1995; Treitel and Carlson 1995). Repressor CreA/CRE1 (CRE: catabolite responsive element) is a homologue of Mig1 in filamentous fungi. Deletion of *CreA*/*CRE1* alleviates CCR‐related cellulolytic enzyme expression (Orejas et al. 1999). Additionally, in *N. crassa* , deficiency of Vib1 reduces the induction of cellulase genes but the expression of CCR‐related genes is increased (Xiong et al. 2014). In *S. cerevisiae* , Snf1 (sucrose nonfermenting 1) phosphorylates Mig1 under glucose‐limited conditions, which relieves the fungus from CCR (Treitel et al. 1998). As a key kinase in the glucose derepression pathway, Snf1 has a vital role in the growth and pathogenesis of plant‐pathogenic fungi (Islam et al. 2017; Lengyel et al. 2022; Yi et al. 2008) by regulating the expression of cell wall‐degrading enzyme (CWDE) genes. Carbon sources may also serve as regulators of gene expression during pathogen infection (Fernandez et al. 2012; Tonukari et al. 2000).

The plant cuticle and cell wall, the native barrier to infection, is composed of cutin, cellulose and pectin (Popper et al. 2011; Ziv et al. 2018). The majority of pathogens that penetrate and thrive in plant cells depend on releasing cutinase (CU), cellulase (CL), pectin lyase (PL) and polygalacturonase (PG) to degrade and weaken the plant cell wall to facilitate the spread of invasive hyphae. *Botrytis cinerea* secretes endopolygalacturonase (BcPG1) for full virulence; SlFERL ( *Solanum lycopersicum* FERONIA Like) interacts with BcPG1 and fine‐tunes MAPK signalling to participate in the immune responses to *B. cinerea* invasion (Ji et al. 2023). *Phytophthora sojae* produces pectin methylesterase (PsPME1) to decrease pectin methylesterification and cooperate with PsPG1 to weaken plant cell walls (Xia et al. 2024). SsPG1 produced by *S. sclerotiorum* is important for infection establishment and is repressed by glucose (Dallal Bashi et al. 2012). However, how pathogens sense and signal environmental conditions to coordinate the expression of hydrolytic enzymes and cell differentiation is not well characterised.

Therefore, we undertook this study to determine whether Ndt80 family TFs regulate carbon metabolism via CCR and to understand how Ndt80 TFs and Snf1 impact CWDEs in *S*. *sclerotiorum*. Here we showed that SsNdt80 TFs have different roles in the development of *S*. *sclerotiorum* and demonstrated that SsNdt80b is essential for nutrient adaptability. Our findings support a function of SsNdt80b in maintaining the optimal expression of *SsSNF1* and modulating the expression of CWDEs during growth and infection by *S*. *sclerotiorum*.

## 2 Results

### 2.1 Ndt80 DNA‐Binding Domain Family TFs Have Different Roles in Sclerotia Formation of *S. Sclerotiorum*

**FIGURE 1:**
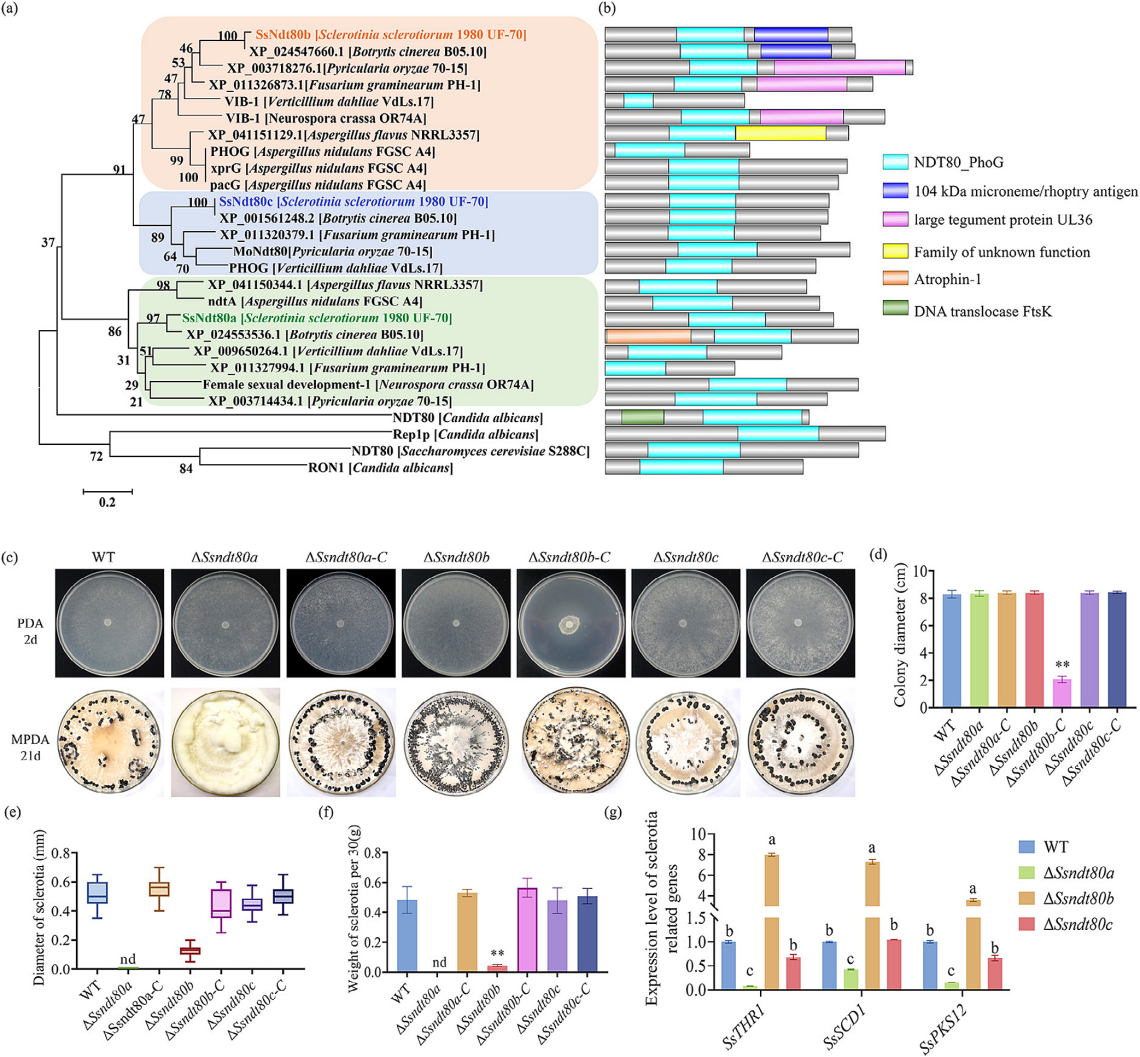
Ndt80 family transcription factors regulated sclerotia formation and hyphal fusion of *Sclerotinia sclerotiorum*. (a) Phylogenetic tree of Ndt80 family transcription factors. The accession numbers and sequences of these proteins are listed in Table S1. (b) Distribution of conserved domains in Ndt80 family transcription factors. Conserved domains were analysed by NCBI (https://www.ncbi.nlm.nih.gov) and Interpro (https://www.ebi.ac.uk/interpro/), then visualised by GPS 2.0 software. (c) Morphology of wild type (WT), *SsNDT80* mutants and complemented strains. Colonies were cultured on potato dextrose agar (PDA) and photographs were taken after 2 days. Sclerotia formation of WT, *SsNDT80* mutants and complemented strains on mashed potato dextrose agar (MPDA) and photographs were taken after 21 days. (d) Colony diameter of WT, *SsNDT80* mutants and complemented strains. Significant difference, **p < 0.01. (e) Sclerotial diameter of WT, *SsNDT80* mutants and complemented strains. nd, not determined. (f) Weight of 30 sclerotia. nd, not determined. Significant difference, **p < 0.01. (g) The expression level of genes related to sclerotial development in WT and *SsNDT80* mutants. Student's *t* test was used to determine statistical significance; different letters mean a significant difference at α = 0.05.

The Ndt80 DNA‐binding domain was used to seek homologous genes in the *S. sclerotiorum* genome sequence; three Ndt80 transcription factors (Sscle_11g085430, Sscle_03g025080 and Sscle_02g019820) were identified in *S. sclerotiorum*. Evolutionary tree analysis showed that these three Ndt80 TFs were in different branches, and most of them have not been previously reported. We named Sscle_11g085430, Sscle_03g025080 and Sscle_02g019820 as SsNdt80a, SsNdt80b and SsNdt80c, respectively (Figure 1a). We analysed the conserved domains in these proteins (Figure 1b). The distribution of conserved domains varies among fungal species, suggesting potential functional divergence among Ndt80 TFs. However, further experimental validation is required.

For further examination of the biological function of SsNdt80s in *S. sclerotiorum*, the hygromycin resistance gene (hygromycin phosphotransferase, *hph*) was used to replace the target genes, and *SsNDT80* mutants were constructed (Figure S1). Complemented strains were created by transforming pNAH‐ONG‐*SsNdt80*s into the *SsNDT80* mutants. Δ*Ssndt80a*, Δ*Ssndt80b* and Δ*Ssndt80c* had a normal mycelial growth rate on potato dextrose agar (PDA) (Figure 1c). However, the colony diameter of the complemented strain Δ*Ssndt80b‐C* was reduced significantly, so that we initially considered that *SsNDT80b* may be involved in the regulation of hyphal growth (Figure 1c,d). Moreover, significant differences in sclerotia formation were observed among Δ*Ssndt80a*, Δ*Ssndt80b* and Δ*Ssndt80c* (Figure 1c): Δ*Ssndt80a* abolished sclerotia formation, Δ*Ssndt80b* developed numerous small abnormal sclerotia that gathered in sheets, while the sclerotia of Δ*Ssndt80c* were no different from the wild type (WT) (Figure 1e,f). Although Δ*Ssndt80b* produced malformed sclerotia, it did not affect their germination to produce hyphae. *SsTHR1* (trihydroxynaphthalene reductase 1), *SsSCD1* (scytalone dehydratase 1) and *SsPKS12* (polyketide synthase 12) are genes related to the development and melanin synthesis of sclerotia in *S. sclerotiorum* (Jiao et al. 2022; Liang et al. 2018; Liang and Rollins 2018). When the expression of sclerotial development genes was compared between the WT and *SsNDT80* mutants, the expression levels of *SsTHR1*, *SsSCD1* and *SsPKS12* were all significantly decreased in Δ*Ssndt80a*, upregulated in Δ*Ssndt80b* and had no significant difference in Δ*Ssndt80c* (Figure 1g). The results of gene expression were consistent with the phenotype. The above results suggested that Ndt80 family TFs play different roles in mycelial growth and sclerotial development in *S. sclerotiorum*: *SsNDT80a* positively regulates sclerotial development, *SsNDT80c* is not different from the WT, while *SsNDT80b* negatively regulates mycelial growth and sclerotia formation.

### 2.2 SsNdt80b Negatively Regulated Pathogenicity of *S. sclerotiorum*

**FIGURE 2:**
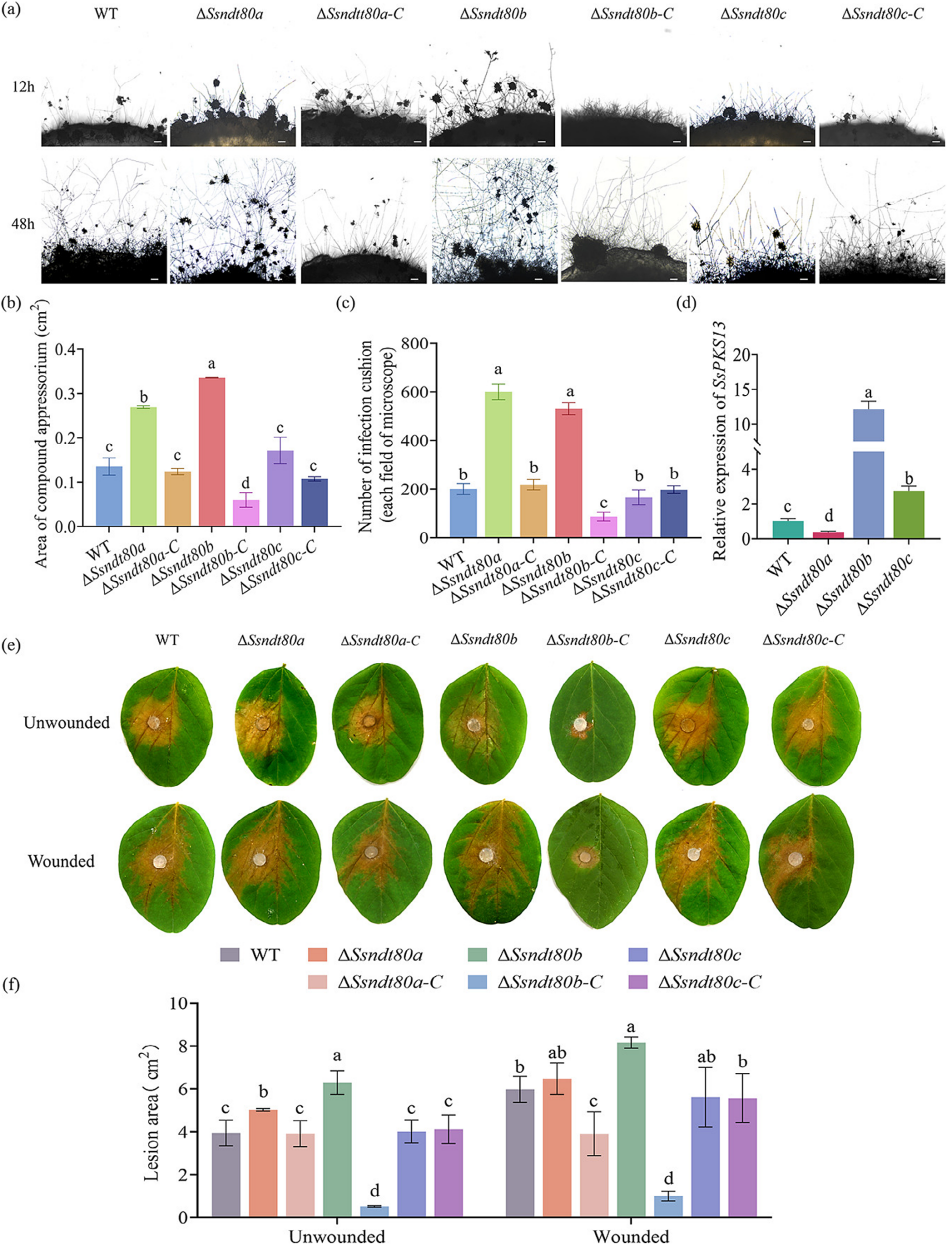
SsNdt80b negatively regulates the development of infection cushions and pathogenicity in *Sclerotinia sclerotiorum*. (a) The morphology of infection cushions of wild type (WT), Δ*Ssndt80a*, Δ*Ssndt80b*, Δ*Ssndt80c* and the complemented strains. Scale bars = 200 μm. (b) The area of infection cushions. ImageJ was used to measure the area of infection cushions of the strains on glass slides after 48 h. (c) Number of infection cushions. The number of infection cushions was counted under a microscope after 48 h. (d) Expression of *SsPKS13* in WT, Δ*Ssndt80a*, Δ*Ssndt80b*, Δ*Ssndt80c* and the complemented strains. The strains were inoculated on glass slides, and infection cushions were collected after 2 days. Total RNA was extracted and *Actin* was used as housekeeping gene to assay the expression level of *SsPKS13*. (e, f) The pathogenicity of WT, Δ*Ssndt80a*, Δ*Ssndt80b*, Δ*Ssndt80c* and the complemented strains. Photographs were taken after 2 days on soybean leaves, and ImageJ was used to measure the lesion area. Different letters above bars indicate significant difference by Student's *t* test at α = 0.05.

In addition to sclerotia, infection cushions are also important infection structures (Liang et al. 2018) of *S. sclerotiorum*. WT and *SsNDT80* mutants as well as complemented strains were inoculated onto glass slides to induce infection cushion development. There was no significant difference in morphology of infection cushions between Δ*Ssndt80a*, Δ*Ssndt80b*, Δ*Ssndt80c* and the WT; however, the formation of infection cushions of Δ*Ssndt80b‐C* was delayed (Figure 2a). Δ*Ssndt80a* and Δ*Ssndt80b* produced more infection cushions than the WT, but Δ*Ssndt80b‐C* produced fewer (Figure 2b,c). *SsPKS13* (polyketide synthase 13) regulates melanin synthesis of infection cushions in *S. sclerotiorum*, and melanin synthesis of infection cushions can affect their development (Li et al. 2018). *SsPKS13* in Δ*Ssndt80b* was significantly upregulated compared to other strains (Figure 2d). This result indicated that *SsNDT80b* negatively regulates the development of infection cushions and the expression of melanin genes.

The pathogenicity on soybean of WT, *SsNDT80* mutants and complemented strains was examined by inoculation on soybean leaves. As shown in Figure 2e,f, the lesion area of Δ*Ssndt80a* and Δ*Ssndt80c* was not different from the WT; however, the lesion area was significantly increased in Δ*Ssndt80b* strains compared to WT on wounded and unwounded soybean leaves, and the virulence of Δ*Ssndt80b‐C* was significantly reduced. These results suggest that *SsNDT80b* is probably involved in the development and pathogenicity of *S. sclerotiorum*.

### 2.3 SsNdt80s Were Involved in Carbon Source Utilisation of *S. sclerotiorum*

**FIGURE 3:**
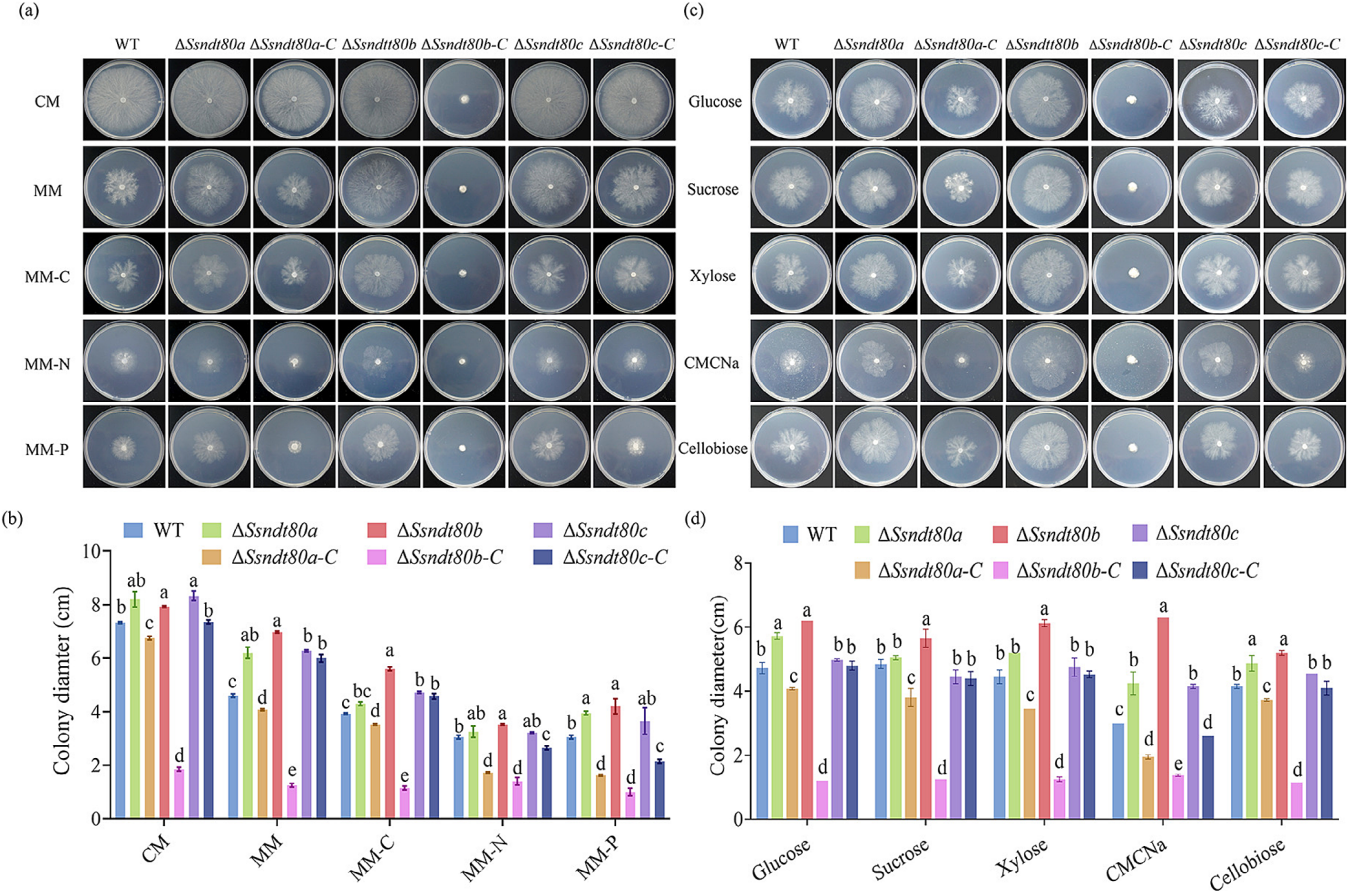
*SsNDT80b* involved in carbon catabolite repression of *Sclerotinia sclerotiorum*. (a) Morphology of wild type (WT), *SsNDT80* mutants and complemented strains on different nutritional media. CM, complete medium; MM, minimal medium; MM − C, −N, −P, minimal medium depleted in carbon, nitrogen or phosphorus, respectively. Pictures were taken after 48 h. (b) Colony diameter of WT, *SsNDT80* mutants and complemented strains on different nutritional media. Different letters indicate significant difference by Student's *t* test at α = 0.05. (c) Growth of the WT, *SsNDT80* mutants and complemented strains on carbon‐replacement media. MM containing glucose was used as the control; in other media, glucose was replaced with the same amount of sucrose, xylose, carboxymethyl cellulose sodium (CMCNa) or cellobiose. Pictures were taken after 48 h. (d) Colony diameter of WT, *SsNDT80* mutants and complemented strains on carbon‐replacement media. Different letters indicate significant difference by Student's *t* test at α = 0.05.

Growth was generally reduced in all strains, including the WT, on minimal medium (MM) compared to complete medium (CM) (Figure 3a,b). Growth on CM, Δ*Ssndt80a* (8.20 ± 0.28 cm), Δ*Ssndt80b* (7.93 ± 0.04 cm) and Δ*Ssndt80c* (8.33 ± 0.18 cm) was significantly faster than the WT (7.33 ± 0.04 cm). The growth advantage of Δ*Ssndt80c* was restored to WT levels in the complemented strain Δ*Ssndt80c‐C* (7.35 ± 0.07 cm), while growth was significantly reduced in the complemented strains Δ*Ssndt80a‐C* (6.75 ± 0.07 cm) and Δ*Ssndt80b‐C* (1.85 ± 0.07 cm). Notably, Δ*Ssndt80b‐C* exhibited the most severe growth reduction compared to WT (mean decrease of 74.8%). Similar trends were observed on MM and under carbon (MM−C), nitrogen (MM−N) and phosphorus (MM−P) depletion conditions. Differences between Δ*Ssndt80a* (6.20 ± 0.21 cm), Δ*Ssndt80b* (6.98 ± 0.04 cm), Δ*Ssndt80c* (6.28 ± 0.04 cm) and WT (4.60 ± 0.07 cm) strains were more pronounced on MM compared to CM; the difference was statistically significant in Δ*Ssndt80b*, with a mean increase of 51.6%. Among all tested conditions, SsNdt80b appeared to have the strongest impact on carbon utilisation, as Δ*Ssndt80b* (5.60 ± 0.07 cm) exhibited the highest growth rate on MM–C, whereas its complement Δ*Ssndt80b‐C* (1.15 ± 0.07 cm) showed a significant reduction in growth compared to WT (3.93 ± 0.04 cm), with a mean decrease of 70.7%. Thus, *SsNDT80b* is probably related to carbon source utilisation. Because Ndt80b TF is homologous to Vib1 TF of *N. crassa* , and Vib1 is involved in CCR (Xiong et al. 2014), we suspect that the phenotype of insensitivity to carbon source deficiency of Δ*Ssndt80b* may be related to CCR.

CCR is the phenomenon whereby pathogens preferentially utilise glucose (a fast‐available carbon source) and activate carbon metabolism repression, inhibiting the utilisation of other carbon sources. Therefore, we used xylose, carboxymethyl cellulose (CMC) and cellobiose as alternative carbon sources to replace glucose as the carbon source in MM. Only Δ*Ssndt80b* had no significant difference in colony diameter between glucose and alternative carbon sources (Figure 3c,d), which further proved that *SsNDT80b* may be involved in CCR of *S. sclerotiorum*.

**FIGURE 4:**
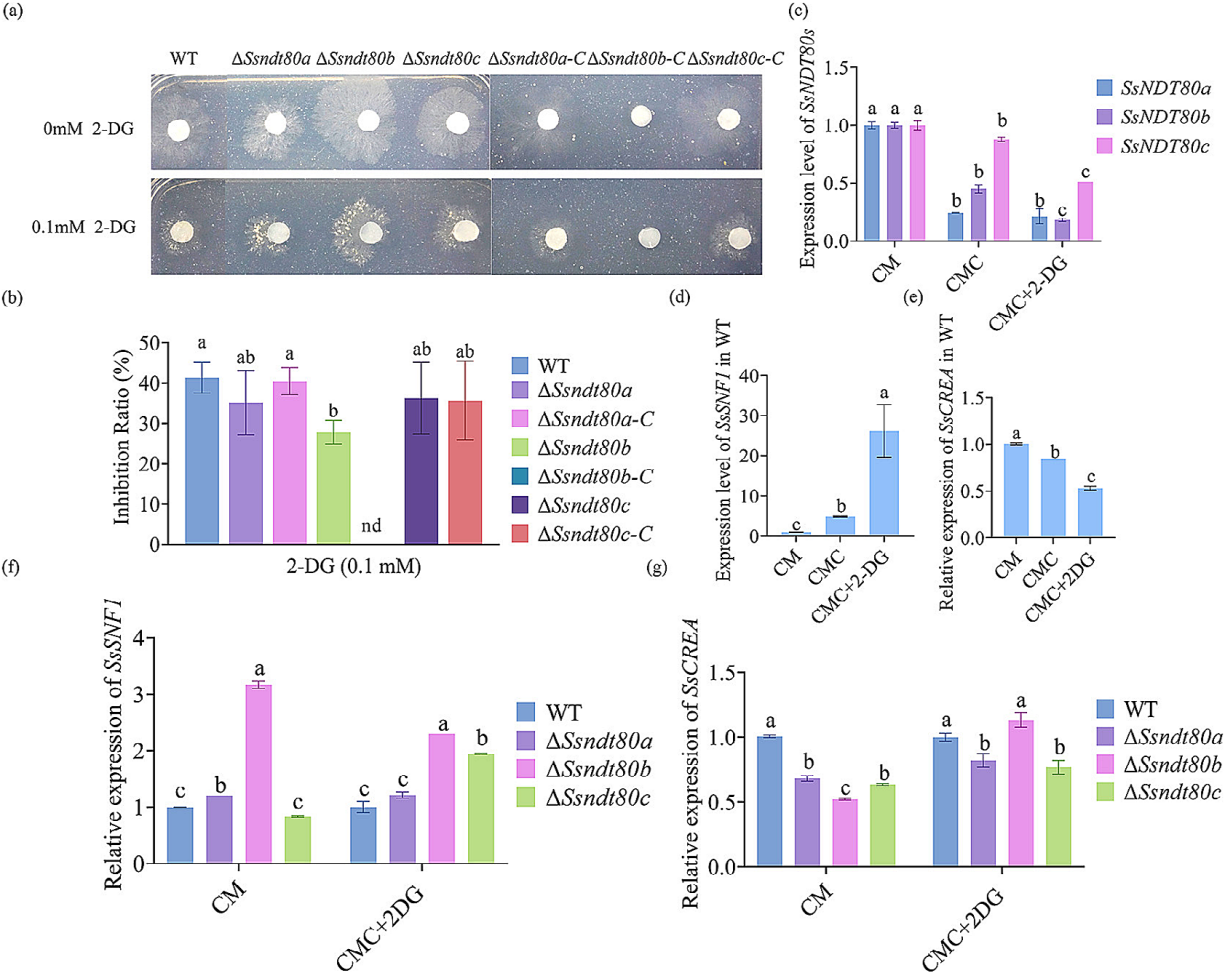
*SsNDT80b* influences the expression of *SsSNF1* and *SsCREA*. (a) *Sclerotinia sclerotiorum* wild type (WT), *SsNDT80* mutants and complemented strains on carboxymethyl cellulose (CMC) medium with or without the nonmetabolisable glucose analogue 2‐deoxy‐d‐glucose (2‐DG). (b) Inhibition ratio of WT, *SsNDT80* mutants and complemented strains on medium containing 2‐DG. (c, d, e) Relative expression of *SsNDT80*s (c), *SsSNF1* (d) and *SsCREA* (e) in *S. sclerotiorum* under carbon catabolite repression conditions. CM, complete medium. (f, g) Deficiency of *SsNDT80*s affected the expression of *SsSNF1* (f) and *SsCREA* (g). The hyphae of WT and *SsNDT80* mutants were cultured in CM for 36 h, transferred into liquid medium containing 0.1 mM 2‐DG for 12 h, and then the hyphae were collected, and total RNA was extracted. Different letters indicate significant difference by Student's *t* test at α = 0.05.

2‐Deoxy‐d‐glucose (2‐DG) is an analogue of glucose that cannot be metabolised. In filamentous fungi, 2‐DG is phosphorylated in functional CCR strains, resulting in the inability of the strain to grow on alternative carbon sources. Thus, 2‐DG can be used to assay the impairment of CCR and glucose repression. We cultured the WT, *SsNDT80* mutants, and complemented strain on medium containing 2‐DG to evaluate whether *SsNDT80*s are involved in CCR and glucose repression. Δ*Ssndt80b* was more insensitive to 2‐DG exposure than the WT (Figure 4a,b), which indicated that the CCR was not functional in Δ*Ssndt80b*. Then, we tested whether *SsNDT80*s could respond to CCR. The relative expression levels of *SsNDT80* genes decreased under 2‐DG treatment, with *SsNDT80b* showing a statistically significant reduction (Figure 4c). These results further demonstrated that *SsNDT80b* is involved in carbon source response and CCR in *S. sclerotiorum*.

*SNF1* and *CREA* are the key genes in the CCR pathway. Therefore, we measured the expression of *SsCREA* (*SS1G_09934*) and *SsSNF1* (Sscle_16g110040) in WT. *SsSNF1* was upregulated under CCR conditions (Figure 4d) whereas the relative expression of *SsCREA* was downregulated under CCR conditions (Figure 4e), which demonstrates the repressive role of *SsCREA* in CCR. We suspected that *SsNDT80*s might influence the expression of *SsCREA* and *SsSNF1*; therefore, we also measured the expression of *SsCREA* and *SsSNF1* among the *SsNDT80* mutants. Notably, in Δ*Ssndt80b*, the expression of *SsSNF1* showed a remarkable increase even in CM (Figure 4f). Additionally, the expression of *SsCREA* was also reduced like that in WT. The results suggest that SsNdt80b is essential for hyphal growth under carbon deficiency and for proper SsSnf1 expression.

### 2.4 SsNdt80b Maintained the Expression of *SsSNF1* in *S. sclerotiorum*

**FIGURE 5:**
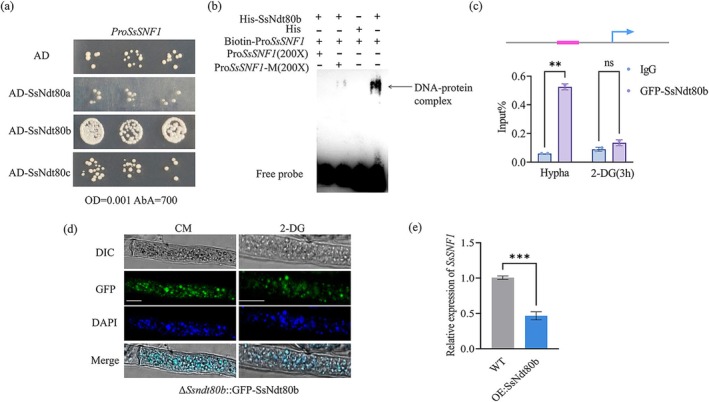
SsNdt80b binds the promoter of *SsSNF1* and inhibits its transcription. (a) Yeast one‐hybrid assays showed that SsNdt80b bound to the promoter of *SsSNF1*. pGADT7 (AD‐emp) was used as a negative control. (b) Electrophoretic mobility shift assay suggesting that SsNdt80b bound to the promoter of *SsSNF1*. The biotin‐labelled probe was the motif, SsNdt80b‐His is the purified fusion protein. Labelled probe with 6x‐His protein was used as the negative control. (c) Chromatin immunoprecipitation‐quantitative PCR assay indicates that SsNdt80b could bind the promoter of *SsSNF1* in *Sclerotinia sclerotiorum*. Student's *t* test was used to determine statistical significance, **p < 0.01, ns *p* > 0.05. (d) SsNdt80b is located in the nucleus. CM, complete medium; 2‐DG, nonmetabolisable glucose analogue 2‐deoxy‐d‐glucose in carboxymethyl cellulose (CMC) medium; DAPI, nucleus stain 4′,6‐diamidino‐2‐phenylindole. (e) Analysis of *SsSNF1* gene expression in wild type (WT) and overexpression strain OE:SsNdt80b. ***p < 0.001 (Student's *t* test).

Analysis of promoter sequence of *SsSNF1* revealed that there is a motif that could bind with members of the Ndt80 family. To determine whether SsNdt80 TFs could bind to the promoter of *SsSNF1*, we constructed pAbAi‐Pro*SsSNF1* and transformed it into yeast cells to produce a bait‐reporter strain. SsNdt80s were subcloned into an AD vector, then a yeast one‐hybrid (Y1H) assay was used to detect binding of SsNdt80s to the promoter of *SsSNF1*. When Pro*SsSNF1* was coexpressed with SsNdt80b, the transformed yeast strains grew well on SD/−Leu/AbA culture plates, indicating that SsNdt80b did bind to the promoter region of *SsSNF1* (Figure 5a). Electrophoretic mobility shift assays (EMSAs) were performed to verify the results of the Y1H assay. SsNdt80b‐His was purified and incubated with a biotin‐labelled DNA probe. Specific SsNdt80b protein–DNA complexes were detected when the labelled DNA probe was used (Figure 5b).

We also used GFP‐tagged SsNdt80b to perform a chromatin immunoprecipitation (ChIP)‐quantitative PCR (qPCR) assay in *S. sclerotiorum*. The subcellular location of GFP‐SsNdt80b was monitored; SsNdt80b was observed in the nucleus under nutrition‐sufficient and ‐deficient conditions (Figure 5d). ChIP‐qPCR assays suggested that the *SsSNF1* promoter accumulated significantly when immunoprecipitated using GFP‐SsNdt80b but not with GFP alone (Figure 5c). An overexpression vector was constructed by cloning *SsNdt80b* into the pNAH‐ONG vector, which contains a strong promoter, and this was then transferred into Δ*Ssndt80b* through protoplast transformation experiments to create OE:SsNdt80b. We measured the expression of *SsSNF1* in OE:SsNdt80b, which was significantly reduced compared to the WT (Figure 5e). These results further suggest that SsNdt80b acts as a transcriptional regulator of *SsSNF1*, inhibiting the expression of *SsSNF1*.

### 2.5 SsSnf1 and SsCreA Are Critical for the Pathogenicity of *S. sclerotiorum*

**FIGURE 6:**
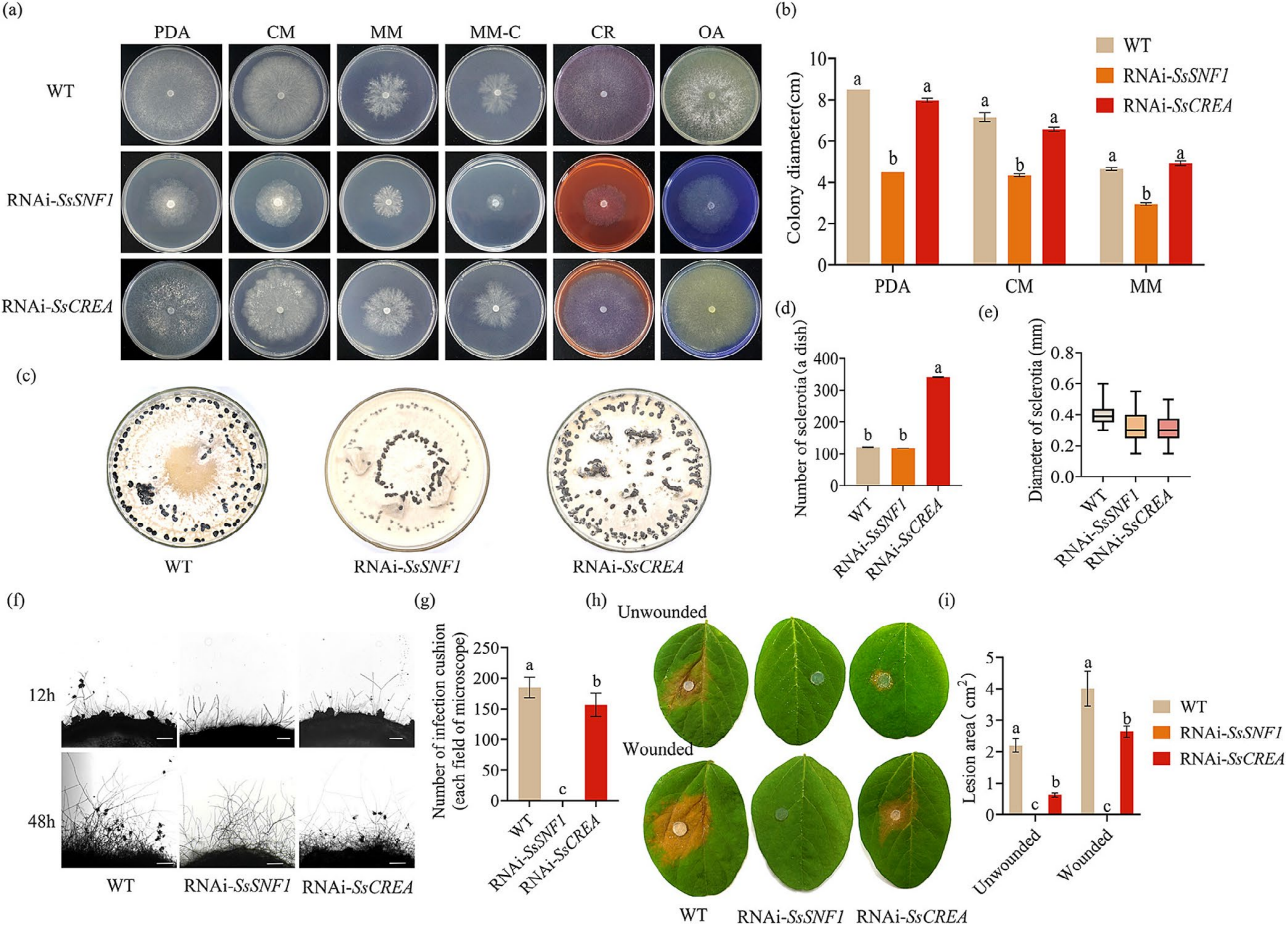
SsSnf1 and SsCreA are important for infection cushion formation and pathogenicity of *Sclerotinia sclerotiorum*. (a, b) Mycelial growth of wild type (WT), RNAi*‐SsSNF1* and RNAi*‐SsCREA* strains on various media. PDA, potato dextrose agar; CM, complete medium; MM, minimal medium; MM−C, minimal medium depleted in carbon; CR, Congo red; OA, oxalic acid. (c) Sclerotia formation of WT, RNAi*‐SsSNF1* and RNAi*‐SsCREA* strain on mashed potato dextrose agar (MPDA). Photographs were taken after 4 weeks. (d) Number of sclerotia for strains grown on MPDA. (e) Diameter of sclerotia of strains on MPDA. (f) Infection cushion development of strains on glass slides. Photographs were taken after 12 and 48 h. (g) Number of infection cushions. ImageJ was used to measure the infection cushions. (h, i) Pathogenicity analysis of strains. Photographs were taken after 2 days on soybean leaves. ImageJ was used to measure the lesion area. Different letters indicate significant difference by Student's *t* test at α = 0.05.

Snf1 is a central element of glucose repression signalling and performs key roles in activating transcription and suppressing gene expression. The CCR protein CreA/CRE‐1 is important in regulating cellular responses to carbon sources. *SsCreA*‐ and *SsSnf1*‐silenced strains were constructed to further examine their biological functions and the responses to carbon sources in *S. sclerotiorum*. The vector pSilent was used to construct silenced strains. Reverse transcription‐quantitative PCR (RT‐qPCR) analysis showed that *SsSNF1* was expressed approximately 60% less in the RNAi*‐SsSNF1* strain and *SsCREA* was expressed about 50% less in the transgenic RNAi*‐SsCREA* strain compared to the WT (Figure S2). The growth of the RNAi*‐SsSNF1* strain was visibly reduced on PDA and barely grew when the carbon source was limited (Figure 6a,b). The sclerotia were smaller (Figure 6c–e) and the development of infection cushions was abolished (Figure 6f,g). The results suggest that SsSnf1 plays a crucial role in the development of *S. sclerotiorum* and the growth defect observed in OE:SsNdt80b may result from reduced *SsSNF1* expression.

Because CreA has been shown to be a key suppressor of CCR, we also analysed the function of SsCreA in *S. sclerotiorum*. Unlike SsSnf1, SsCreA did not affect growth (Figure 6a,b) or sclerotia (Figure 6c–e). However, the number of infection cushions was significantly reduced (Figure 6f,g). Furthermore, the virulence of RNAi*‐SsSNF1* and RNAi*‐SsCREA* was reduced (Figure 6h,i). The results suggest that the proper functioning of CCR is important for the growth and development of *S. sclerotiorum*.

### 2.6 SsNdt80b Impacted the Production of CWDEs in *S. sclerotiorum*

**FIGURE 7:**
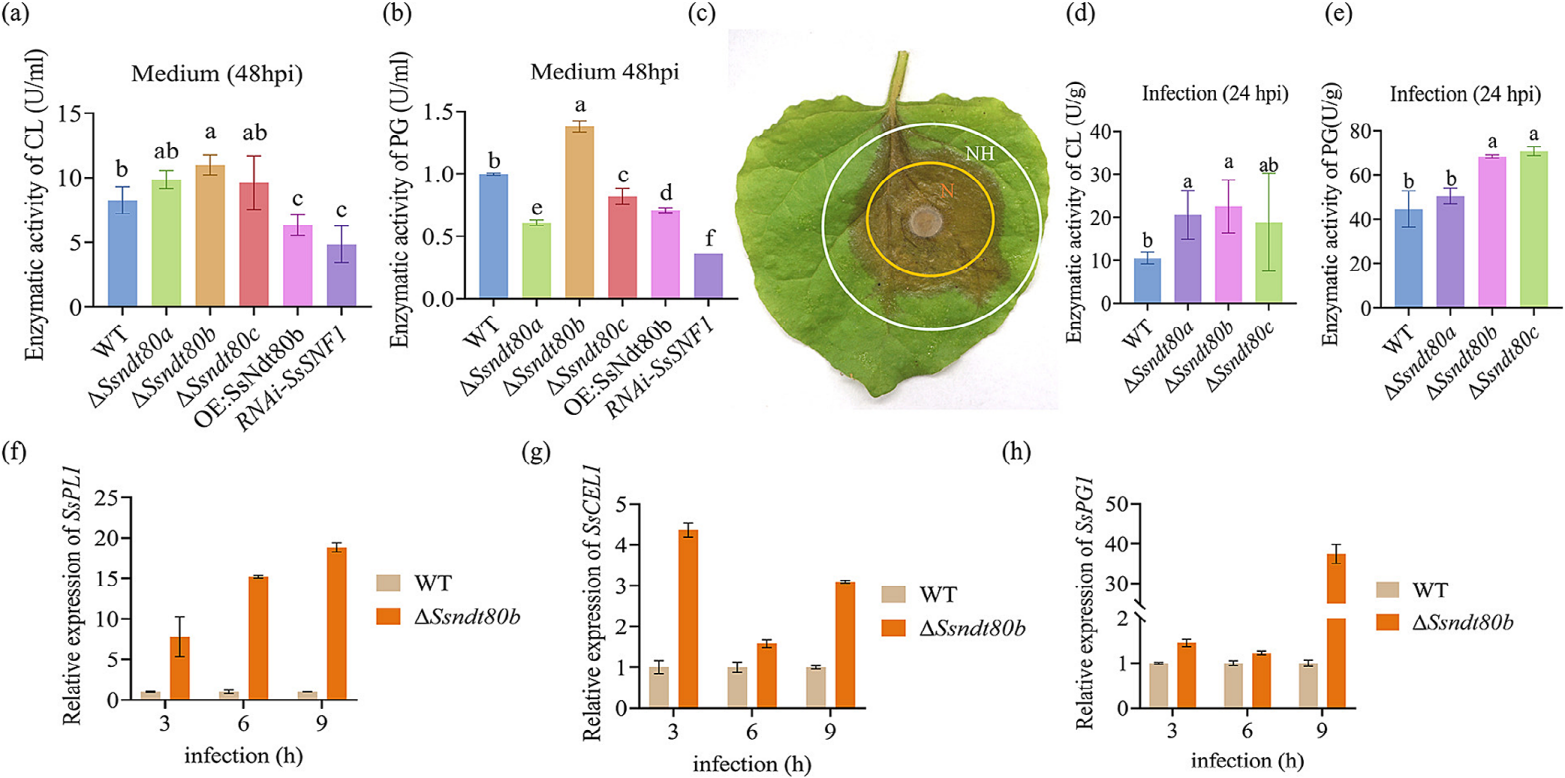
SsNdt80b negatively regulated SsSnf1 and affected the production of cell wall‐degrading enzymes (CWDEs) in *Sclerotinia sclerotiorum*. (a, b) The cellulase (CL) and polygalacturonase (PG) enzyme activity of wild type (WT), *SsNDT80* mutants, overexpression strain OE:SsNdt80b and RNAi‐*SsSNF1* strains in the liquid medium for inducing cell wall‐degrading enzymes at 48 h postinoculation (hpi). (c) Photograph showing the junction of diseased (N) and healthy (NH) *Nicotiana benthamiana* leaf tissue. (d, e) The CL and PG enzyme activity of WT and *SsNDT80* mutants on a leaf. Different letters indicate significant difference by Student's *t* test at α = 0.05. (f, g, h) The relative expression levels of CWDE genes in the WT and *SsNDT80b* mutant during infection.

Cellulolytic enzyme production requires proper nutrient sensing and relief from CCR. To test whether the increased pathogenicity of SsNdt80b was related to the CWDEs, we compared the CL and PG enzyme activity between WT, *SsNDT80* mutants, OE:SsNdt80b and RNAi‐*SsSNF1* strains. In the liquid induction medium, the activities of CL and PG in OE:SsNdt80b and RNAi‐*SsSNF1* strains were significantly lower than those of the WT, and the Δ*Ssndt80b* showed increased enzyme activity compared to the WT (Figure 7a,b). CWDEs are remarkably induced during the infection phase; thus, we performed enzyme activity analysis at the junction between healthy and diseased tissue after inoculation (Figure 7c). As shown in Figure 7d,e, Δ*SsNdt80b* also showed high levels of CL and PG enzyme activity. The results indicate that *SsNDT80b* may affect the activity of CL and PG by regulating the expression of *SsSNF1*.

We also measured the relative expression of *SsPL1* (pectate lyase, *SS1G_00238*), *SsCEL1* (cellulase, *SS1G_00458*) and *SsPG1* (polygalacturonase, *SS1G_10167*) in WT and Δ*SsNdt80b*, which are important CWDEs during the infection by *S. sclerotiorum*. In Δ*SsNdt80b*, *SsPG1*, *SsCEL1* and *SsPG1* were all upregulated during the infection process (Figure 7f–h). These results indicated that SsNdt80b negatively regulated SsSnf1, modulating the production of CWDEs in *S. sclerotiorum*.

## 3 Discussion

**FIGURE 8:**
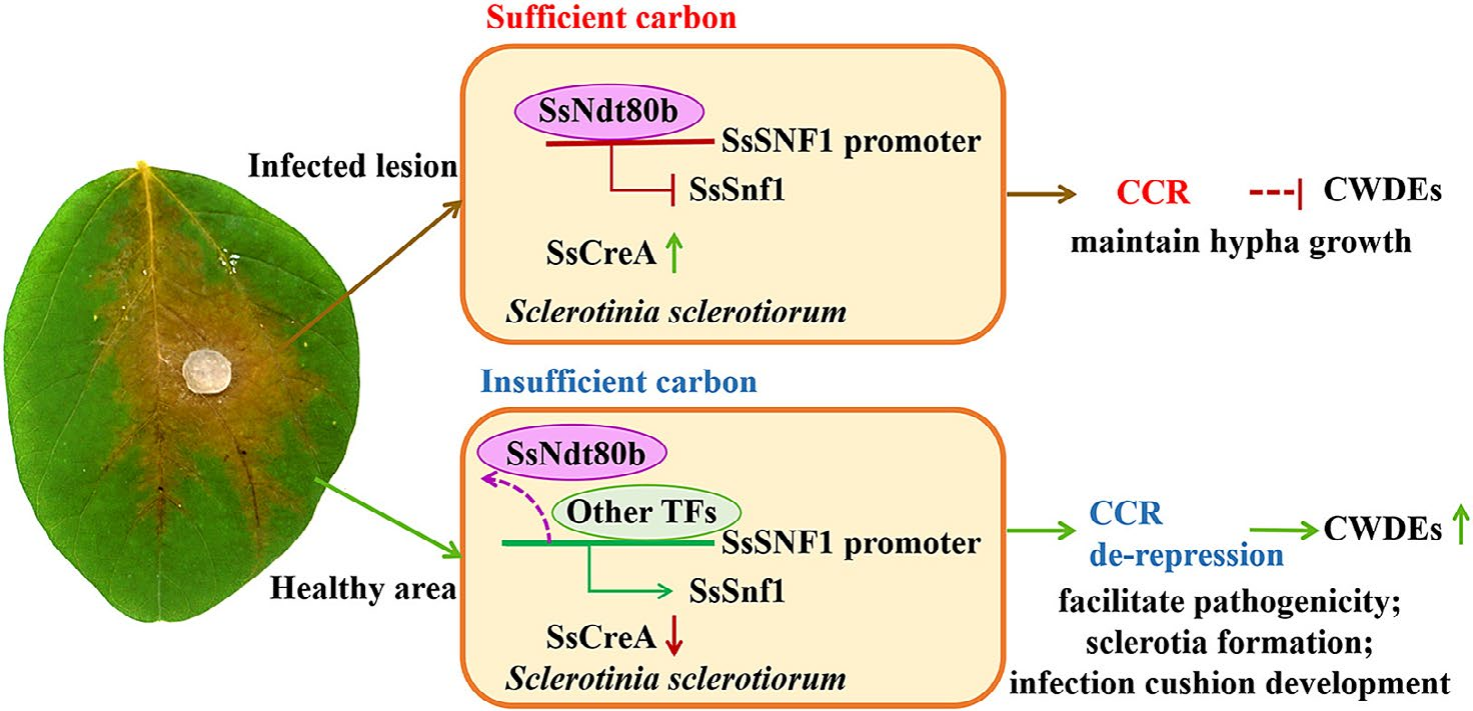
Model of how SsNdt80b responds to carbon source and modulates SsSnf1 to regulate the development and pathogenicity of *Sclerotinia sclerotiorum*. CCR, carbon catabolite repression; CWDE, cell wall‐degrading enzyme; TF, transcription factor.

*Sclerotinia sclerotiorum* is a representative necrotrophic phytopathogenic fungus; the primary source of nutrients for its growth is dead plant biomass. In this research, we determined that Ndt80 family TFs are connected with carbon source utilisation and have different roles in the growth, sclerotia formation, infection cushion development and the virulence of *S. sclerotiorum*. SsNdt80b responded to limited carbon sources and removed the expression restriction of SsSnf1, which upregulated the production of CWDEs and facilitated the pathogenicity of *S. sclerotiorum* (Figure 8).

The Ndt80 TF, containing the conserved NDT80_PhoG domain, exhibits functional diversity across fungal species. Initially identified in *S. cerevisiae* for its role in meiotic nuclear division, Ndt80 homologues perform distinct regulatory functions in different fungi. In *Aspergillus nidulans*, XprG (PhoG) regulates the carbon starvation response and conidial development (Katz et al. 2013). In *N. crassa* , *VIB1* is involved in heterokaryon incompatibility, programmed cell death and CCR (Xiong et al. 2014). In *Trichoderma reesei*, *RON1* serves as a key activator of the *N*‐acetylglucosamine (*GlcNAc*) gene cluster, linking it to GlcNAc metabolism (Kappel et al. 2016). In plant‐pathogenic fungi, MoNdt80 in *Magnaporthe oryzae* regulates GlcNAc catabolism and CWDEs, contributing to virulence (Bhatt et al. 2020). In the human fungal pathogen *C. albicans* , CaNdt80 is essential for antifungal resistance, hyphal growth and pathogenicity, with *REP1* and *RON1* playing distinct roles in virulence regulation (Chen et al. 2004). Given its critical role in fungal development and pathogenesis, understanding Ndt80 in *S. sclerotiorum* remains an important area of investigation. Further study may provide valuable insights into sclerotium formation and potential strategies for disease control.

SsNdt80a is homologous with female sexual development‐1 (Fsd1) in *N. crassa* (Figure 1a). The *Fsd1* mutant is defective in female sexual development and ascospore maturation (Hutchison and Glass 2010). Δ*Ssndt80a* abolished sclerotia formation, but did not affect the infection cushion and pathogenicity of *S. sclerotiorum*. SsNdt80b is related to Vib1, but the conserved domain in these proteins is different (Figure 1b), which probably indicates a differential biological function between these proteins. Δ*vib1* shows reduced growth (Xiong et al. 2014); however, Δ*Ssndt80b* exhibited increased growth compared to WT on MM and carbon source‐limited medium, which may be due to the abnormal expression of *SsSNF1* and *SsCREA* in Δ*Ssndt80b*. SsNdt80c differs from SsNdt80a and SsNdt80c: the SsNdt80c mutant had the same phenotype as the WT. These results indicate that the function of Ndt80 family TFs is highly variable.

Glucose is the preferred carbon source for most organisms. Accordingly, genes related to catabolise other carbon sources are repressed by the presence of glucose in a process known as carbon catabolite repression (CCR) (Wu et al. 2020). The availability of glucose can also inhibit the production of CWDEs (Xiong et al. 2014). CCR ensures that genes needed to assimilate carbon sources other than glucose are not strongly expressed; thus, CCR may enhance fitness through energy conservation (Fernandez et al. 2012; Wu et al. 2020). As reported, glucose repression protein kinase Snf1 is a critical protein in the CCR. *CcSNF1* is required for biochemical processes important in pathogenesis by *Cochliobolus carbonum*, which affects the expression of CWDEs (Tonukari et al. 2000). In *Fusarium virguliforme* and *Fusarium oxysporum*, the CWDE‐related genes are also downregulated in *SNF1* mutants (Islam et al. 2017; Ospina‐Giraldo et al. 2003). In *B. cinerea*, xylanase activities showed a twofold reduction after *BcSNF1* gene deletion (Lengyel et al. 2022). *MoSNF1* contributes to growth and pathogenicity in *M. oryzae*, but it does not affect the expression of CWDEs (Yi et al. 2008), which indicates that Snf1 has various functions in fungi. We demonstrated that SsSnf1 was important for the growth, infection cushion formation and pathogenicity of *S. sclerotiorum*, which also affected the CWDE activity. The depression of SsSnf1 in the presence of glucose was related to SsNdt80b.

The C2H2 transcription factors Cre1/CreA in fungi are critical for CCR, with wide roles in diverse physiological processes (Liu et al. 2023; Portnoy et al. 2011). The loss of CreA in *Beauveria bassiana* impacts radical growth, conidiation and blastospore development, antioxidant responses and virulence(Luo et al. 2014). In *Metarhizium robertsii*, Cre1 directly regulates the expression of *hyd4*, a critical hydrophobin gene essential for infection cushion formation and pathogenicity (Lai et al. 2020). CreA promotes *Aspergillus fumigatus* GAG production and biofilm formation by positively regulating the expression of GAG biosynthetic genes (Liu et al. 2023). Studies of *M. oryzae* and *Aspergillus flavus* have suggested that CreA contributes to hyphal growth, asexual development and pathogenicity (Hong et al. 2021). Our results showed that CreA is a negative regulator of sclerotia formation on mashed potato dextrose agar (MPDA) in the presence of glucose, as also seen in *A. fumigatus* where CreA is a negative regulator of asexual development under liquid‐submerged culture conditions (Liu et al. 2023).

When glucose is lacking, fungi must utilise alternative carbon sources to maintain growth and generate energy (Chen et al. 2018). CCR and glucose inhibition must be mitigated to allow assimilation of these alternative carbon sources. The colony diameter of Δ*Ssndt80b* on MM and carbon source‐replaced medium was significantly larger than that of WT, which may be due to SsNdt80b causing relief of the CCR, which enhanced the ability for nutrient assimilation. This process of inhibition may be essential in the initial stage of pathogen infection in plants, when glucose is lacking outside the plant. CWDEs in fungi are often subject to CCR at the transcriptional level (Fernandez et al. 2012). The expression levels of CWDEs, along with many other genes, are downregulated when glucose is sufficient, and one of the main regulators controlling this process is Snf1 (Islam et al. 2017). In phytopathogenic fungi, Snf1 is needed to relieve this repression and to regulate the expression of CWDEs during the infection process (de Oliveira Silva et al. 2024; Islam et al. 2017; Lengyel et al. 2022; Yu et al. 2014).

The production of CWDEs is controlled by transcriptional regulators, whose activity is influenced by nutrients available in the environment. Sensing of plant cell wall polymers or their derivatives induces production of CWDEs, as demonstrated for *Colletotrichum* and *Botrytis* species (de Oliveira Silva et al. 2024; Ji et al. 2023). In *S. sclerotiorum*, CWDEs are indispensable virulence factors that contribute to pathogenicity (Dallal Bashi et al. 2012). SsNdt80b could regulate the expression of *SsSNF1* and influence the production of CL and PG; however, whether SsNdt80b directly modulates other CWDEs needs to be explored further. Taken together, this research supports a function of Ndt80s TFs and SsSnf1 in *S. sclerotiorum*, with SsNdt80b modulating the transcriptional activity of *SsSNF1*, which influences the expression of CWDEs, thus enabling a suitable cellular response for plant tissue destruction and nutrition utilisation and contributing to the growth and pathogenicity of *S. sclerotiorum*.

## 4 Experimental Procedures

### 4.1 Strains and Culture Conditions

*Sclerotinia sclerotiorum* UF‐1 was used as the WT strain to construct gene deletion mutants and GFP‐/RFP‐labelled strains. All strains were cultured on potato dextrose agar (PDA) plates at 22°C for 48 h. For sclerotia production, strains were grown on MPDA (mashed potato dextrose agar: 300 g/L potato, 15 g/L agar) at 22°C for 21 days.

### 4.2 Construction of Gene Deletion, Gene Silencing and Complementation Mutants

NDT80 family deletion mutants were constructed through split‐marker PCR. Briefly, NDT80sF1/NDT80sR1 and NDT80sF2/NDT80sR2 amplified 5' and 3' flanking sequences of *NDT80* genes, NDT80sF1/NLC37 and NLC38/NDT80sR2 were used to fuse the flanking sequences and hygromycin resistance gene cassette. This was then transformed into the WT strain by using polyethylene glycol (PEG)‐mediated protoplast transformation. Hygromycin B (100 μg/mL) was used to select transformants. For gene silencing, the cDNA fragment of *SsSnf1* was amplified with pSD1‐SsSnf1 F1/pSD1‐SsSnf1 R1 and subcloned into the pSilent‐Dual1 vector. pSilent‐Dual1‐SsSnf1 was then transformed into protoplasts of WT, and Neo (100 μg/mL) was used to select silenced transformants. Plasmid pNAN‐GFP was used for the overexpression of genes. All strains were preserved at 4°C. Primer sequences are in Table S1.

### 4.3 Pathogenicity and Infection‐Related Morphogenesis Assays

The infection cushion formation was induced on glass slides with mycelial plugs (7 mm diameter) from WT, *SsNDT80* mutants and the complemented strains Δ*Ssndt80‐C*; then, the inoculated glass slides were put in a humid box at 25°C and photographed after 12, 24 and 48 h. The area of the infection cushion was calculated by ImageJ. Pathogenicity of WT, *SsNdt80* mutants, and the complemented strains Δ*Ssndt80‐C* was assessed by inoculation of fresh mycelial plugs (5 mm diameter) from strains onto leaves of soybean (Williams 82). Disease lesions were photographed 2 days postinoculation (dpi) and the lesion area was calculated by ImageJ. Every experiment has three biological replicates.

### 4.4 Subcellular Location Observation

To construct the plasmids of GFP‐SsNdt80s, full‐length *SsNdt80* genes were amplified and inserted into pNAN‐ONG and transformed into Δ*Ssndt80* strains. To confirm the expression of the GFP fusion proteins, hyphae were cultured on PDA for 24 h, and GFP signals were observed and photographed using Stellaris 5 microsystem (CMS Gmbh; Leica). 4',6‐diamidino‐2‐phenylindole (DAPI; 1 μg/mL; Solarbio) was used to stain the nuclei of hyphae.

### 4.5 Yeast One‐Hybrid Assay

The full‐length of *SsNdt80* cDNA was cloned into pGADT7, the promoter of *SsSNF1* was amplified and inserted into the pAbai vector. Then, pGADT7‐SsNdt80s and pAbai‐ProSsSNF1 were cotransformed into Y1HGOLD and to test the binding ability on SD−Leu containing aureobasidin A (AbA).

### 4.6 Electrophoretic Mobility Shift Assay

For the electrophoretic mobility shift assay (EMSA), we first obtained purified SsNdt80b‐His recombinant protein according to the instructions for Ni‐NTA 6FF His Resin (Biotech). The probe sequence with or without 5'‐terminal biotin labelling was 5'‐TGAAGAGCAGCTGG AACCACAAATCAACTTTTCCTTTAA‐3'. EMSA was performed with SsNdt80b‐His recombinant protein and biotin‐labelled probe according to instructions provided in the LightShift Chemiluminescence EMSA Kit (Thermo). In brief, SsNdt80b‐His recombinant protein was incubated in EMSA binding buffer for 5 min in an ice bath, followed by the addition of 1 μL of labelled probe or mutant probe and incubation for 30 min at room temperature. The incubated samples were electrophoresed on a 6% nondenaturing polyacrylamide gel and then transferred to an Amersham Hybond‐N^+^ membrane. Membranes were analysed using the Tanon imaging system.

### 4.7 Chromatin Immunoprecipitation‐qPCR Analyses

ChIP‐qPCR was applied as published (Xia et al. 2024). In summary, hyphal samples (2 g) were cross‐linked with buffer A (1% formaldehyde, 10 mM Tris–HCl, 1 mM EDTA, 137 g/L sucrose, 1 mM PMSF) and the reaction was stopped with glycine. Subsequently, samples were suspended in extraction buffer with protease inhibitor cocktail (HY‐K0010‐1; MedChemExpres) and centrifuged; nuclei lysis buffer was added, and DNA was broken in an ultrasonic cell disruptor. After centrifugation, the supernatant was divided into three aliquots: input, immunoprecipitation (IP) and mock, which were diluted with 10 × ChIP dilution buffer. Anti‐GFP affinity beads 4FF were added into IP samples; anti‐IgG1 together with the protein A/G magnetic beads were added into mock samples. The beads were washed and eluted, and DNA was precipitated. Input, IP and mock were quantified by qPCR with specific primers (Table S1).

### 4.8 RT‐qPCR

Hyphae, sclerotia and infection cushions were collected, and TransZol Up Kit (ET111‐01‐V2, Transgen Biotech) was used to extract total RNA. RT‐qPCR (housekeeping gene: *ACTIN*) was performed using PerfectStart Green qPCR SuperMix. Primer information is in Table S1.

### 4.9 CWDE Assays

CL catalyses the conversion of cellulose to reducing sugars; the reducing sugar can react with 3,5‐dinitrosalicylic acid (DNS) to produce 3‐amino‐5‐nitrosalicylate, which is a reddish‐brown substance and has a characteristic peak at 540 nm. CL activity can be calculated by measuring the change in absorbance at 540 nm. PG hydrolyses polygalacturonic acid to produce galacturonic acid, which can also react with DNS. The PG and CL activity of strains in culture and in planta were measured using the Polygalacturonase Assay Kit (bc2660; Solarbio) and the Cellulase Assay Kit (bc2540; Solarbio) following the manufacturer's instructions.

Briefly, strains were grown on the surface of cellophane membranes overlaid on PDA. After 2 days, the cellophane membranes with the mycelium were transferred to 15‐mL liquid medium for inducing CWDEs (KNO_3_ 2 g, KCl 0.5 g, FeSO_4_ 0.01 g, K_2_HPO_4_ 1 g, MgSO_4_·7H_2_O 0.5 g, VB1 0.2 mg, L‐asparagine 0.5 g, carboxymethyl cellulose 10 g, 1 L, pH 6.0). The supernatant of each culture was collected at 4 dpi; after centrifugation (12,000 *g*, 10 min), 50 μL osupernatant was used to measure the activity of PG and CL.

For the infected tissue, strains were inoculated on *N. benthamiana*. The infected and healthy margin (0.05 g) was harvested at 48 hpi for protein extraction with the extraction buffer from the kit; the supernatant was collected to measure the activity of PG and CL. EnSight Multimode Plate Reader (PerkinElmer) was used to monitor the reaction at 540 nm. One unit of CL activity is defined as the catalysis of 1 μg glucose per minute per gram of tissue in the reaction system. One unit of PG enzyme activity is defined as the decomposition of polygalacturonic acid per gram sample per hour to produce 1 μmol of galacturonic acid.

### 4.10 Data Analysis

The phylogenetic trees of SsNdt80a, SsNdt80b and SsNdt80c were constructed using the software MEGA 7, and the developmental trees were created by neighbour‐joining analysis by NCBI. InterPro (https://www.ebi.ac.uk/interpro/) was used to analyse the conserved domains and visualise with GPS 2.0 software.

ImageJ was used to measure infection cushions of the strains on glass slides and the lesion area. Student's *t* test was used to determine statistical significance at *p < 0.05, **p < 0.01, ***p < 0.001.

## Conflicts of Interest

The authors declare no conflicts of interest.

## Supporting information


**FIGURE S1.** Verification of *SsNDT80* mutants. (a) Schematic diagram of split‐marker PCR to obtain *SsNDT80* mutants. (b) PCR validation of the *SsNDT80a* mutant and gene expression analysis of *SsNDT80a* in UF‐1, ∆*Ssndt80a* and ∆*Ssndt80a‐C*. (c) PCR validation of the *SsNDT80b* mutant and gene expression analysis of *SsNDT80b* in UF‐1, ∆*Ssndt80b* and ∆*Ssndt80b‐C*. (d) PCR validation of the *SsNDT80c* mutant and gene expression analysis of *SsNDT80c* in UF‐1, ∆*Ssndt80c* and ∆*Ssndt80c‐C*.

**FIGURE S2.** Silencing *SsSNF1* and *SsCREA* in UF‐1. (a) Schematic diagram to obtain *SsSNF1* and *SsCREA* silencing strains. (b, c) Gene expression analysis of *SsSNF1* and *SsCREA* in UF‐1 and gene silencing strains.

**Table S1.** Primer information of this study.

## Data Availability

The data that support the findings of this study are available on request from the corresponding author.
