# Supplementary material for: Transcription Factor SsNdt80b Maintains Optimal Expression of SsSNF1 to Modulate Growth and Pathogenicity in Sclerotinia sclerotiorum
Source: Mol Plant Pathol. 2025 Apr 19;26(4):e70088. doi: 10.1111/mpp.70088 (PMC12008772; doi:10.1111/mpp.70088)
Supplement: Supplementary file 1 — FIGURE S1. Verification of SsNDT80 mutants. (a) Schematic diagram of split‐marker PCR to obtain SsNDT80 mutants. (b) PCR validation of the SsNDT80a mutant and gene expression analysis of SsNDT80a in UF‐1, ∆Ssndt80a and ∆Ssndt80a‐C. (c) PCR validation of the SsNDT80b mutant and gene expression analysis of SsNDT80b in UF‐1, ∆Ssndt80b and ∆Ssndt80b‐C. (d) PCR validation of the SsNDT80c mutant and gene expression analysis of SsNDT80c in UF‐1, ∆Ssndt80c and ∆Ssndt80c‐C. [file MPP-26-e70088-s001.docx]

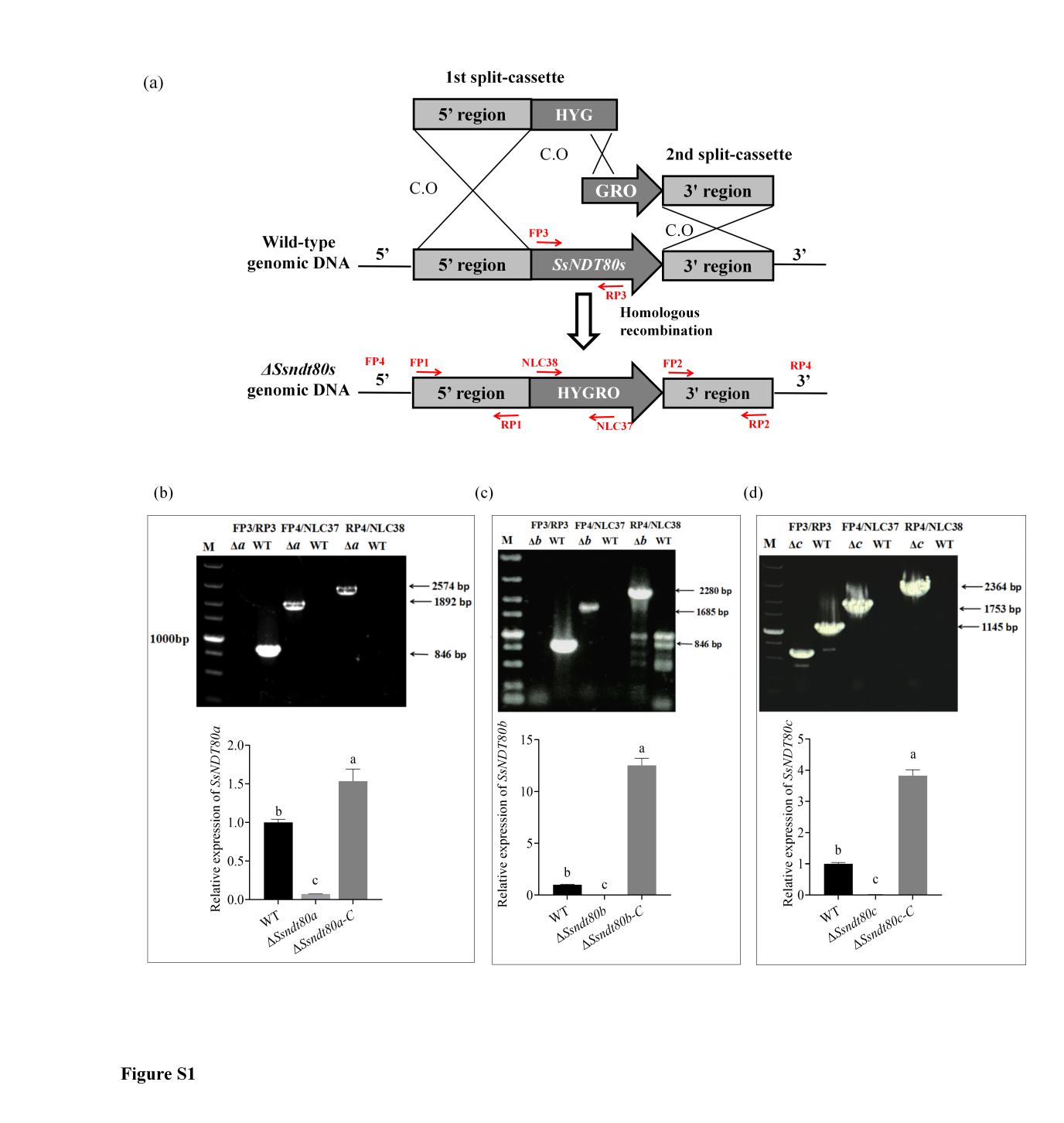


Figure S1 Verification of *SsNDT80s* mutants.

1. Schematic diagram of Split-marker PCR to obtain *SsNDT80s* mutant. (b) PCR validation of the *SsNDT80a s*mutant and gene expression analysis of *SsNDT80a* in UF-1, ∆*Ssndt80a* and ∆*Ssndt80a-C.* (c) PCR validation of the *SsNDT80b* mutant and gene expression analysisof *SsNDT80b* in UF-1, ∆*Ssndt80b* and ∆*Ssndt80b-C*. (d) PCR validation of the *SsNDT80c* mutant and gene expression analysisof *SsNDT80c* in UF-1, ∆*Ssndt80c* and ∆*Ssndt80c-C*.
