# Supplementary material for: Transcription Factor SsNdt80b Maintains Optimal Expression of SsSNF1 to Modulate Growth and Pathogenicity in Sclerotinia sclerotiorum
Source: Mol Plant Pathol. 2025 Apr 19;26(4):e70088. doi: 10.1111/mpp.70088 (PMC12008772; doi:10.1111/mpp.70088)
Supplement: Supplementary file 2 — FIGURE S2. Silencing SsSNF1 and SsCREA in UF‐1. (a) Schematic diagram to obtain SsSNF1 and SsCREA silencing strains. (b, c) Gene expression analysis of SsSNF1 and SsCREA in UF‐1 and gene silencing strains. [file MPP-26-e70088-s002.docx]

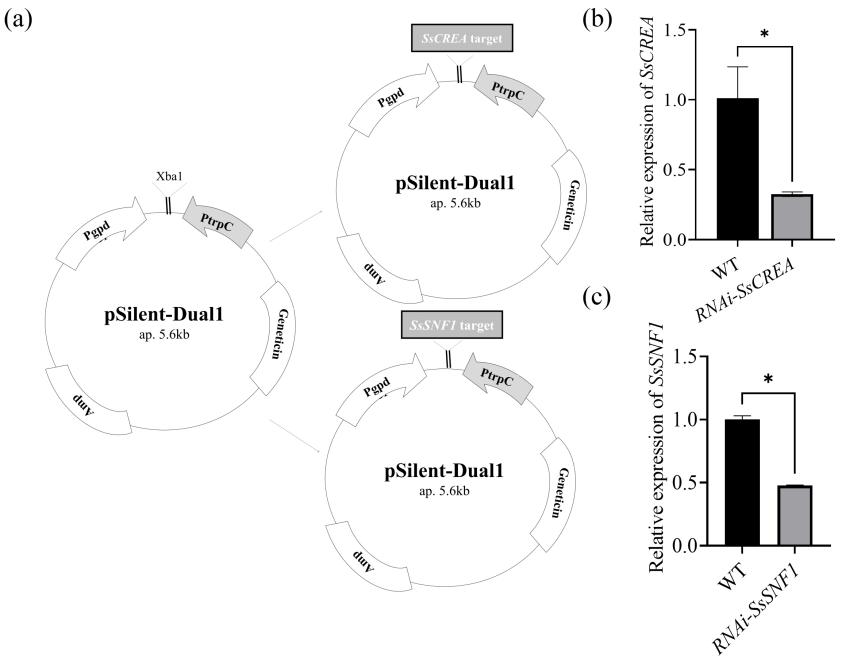


Figure S2 Silencing *SsSNF1* and *SsCREA* in UF-1.

(a) Schematic diagram to obtain *SsSNF1* and *SsCREA* silencing strains. (b, c) Gene expression analysis of *SsSNF1* and *SsCREA* in UF-1 and gene silencing strains*.*
