# Supplementary material for: Transcription Factor SsNdt80b Maintains Optimal Expression of SsSNF1 to Modulate Growth and Pathogenicity in Sclerotinia sclerotiorum
Source: Mol Plant Pathol. 2025 Apr 19;26(4):e70088. doi: 10.1111/mpp.70088 (PMC12008772; doi:10.1111/mpp.70088)
Supplement: Supplementary file 3 — Table S1. Primer information of this study. [file MPP-26-e70088-s003.docx]

Table S1 Primers information of this study.

| Primers | Sequences (5’-3’) |
| --- | --- |
| Ssndt80a-FP1 | TCCTGTGTGAAATTGTTATCCGCTGTATCGGATGATGGAAAA |
| Ssndt80a-RP1 | CGAAATGGTTGGTGTAGT |
| Ssndt80a-FP2 | GTCGTGACTGGGAAAACCCTGGCGCGCATCATAGTCTCATCG |
| Ssndt80a-RP2 | TCAGGTCCGTCACTCTTC |
| Ssndt80a-FP3 | CTGGAATGGCACCACCCT |
| Ssndt80a-RP3 | GGACCGCAACTTTCACCC |
| Ssndt80a-FP4 | GATTTTGGGGGGTGTCGTTG |
| Ssndt80a-RP4 | TCCCCACAAGCCATTTGTCA |
| Ssndt80b-FP1 | ATACTGGTTGGTGCTGAA |
| Ssndt80b-RP1 | TCCTGTGTGAAATTGTTATCCGCTTTGAATGAGTAGAAGTGGC |
| Ssndt80b-FP2 | GTCGTGACTGGGAAAACCCTGGCGATTTCCTCCGATTTACAC |
| Ssndt80b-RP2 | CAAAGCCAAGAAAGAATG |
| Ssndt80b-FP3 | ATAGACTACTTTCCTTCAGC |
| Ssndt80b-RP3 | ACTGCTGTCTCATCAAATGT |
| Ssndt80c-FP1 | AAGTCGGTACTGATGATAC |
| Ssndt80c-RP1 | TCCTGTGTGAAATTGTTATCCGCTATACGGAGACGAGAATAG |
| Ssndt80c-FP2 | GTCGTGACTGGGAAAACCCTGGCGTGGGATTAGCGACTTCAT |
| Ssndt80c-RP2 | GTTCTTCCTGGTTCCTTT |
| Ssndt80c-FP3 | GAGGAGAAGGGACGATGT |
| Ssndt80c-RP3 | GGGAGGACGATTATGAGA |
| Ssndt80c-FP4 | GAGTAAGAAGTAACTTCACA |
| Ssndt80c-RP4 | CTCGAGGATTTTTTTAACAT |
| M13R | AGCGGATAACAATTTCACACAGGA |
| NLC37 | GGATGCCTCCGCTCGAAGTA |
| M13F | CGCCAGGGTTTTCCCAGTCACGAC |
| NLC38 | CGTTGCAAGACCTGCCTGAA |
| pSD1-SsCREA-F | GCTCTAGATGCGAAAAGGCATTCCATCG |
| pSD1-SsCREA-R | GCTCTAGAGTGACACATTCGGGCTACCA |
| pSD1-SsSNF1-F | GCTCTAGACGATAGCGCCTCATGCTAGT |
| pSD1-SsSNF1-R | GCTCTAGACGCTCCTTGACAAGAGGTGA |
| ONG-Ssndt80a-F | GAATGGATGAACTTTACAAAATGGGGATGCGATATTTGAT |
| ONG-Ssndt80a-R | CATCTTATCTACATACGCTACTAGCTGATATTAAGCTCCTGCG |
| ONG-Ssndt80b-F | GAATGGATGAACTTTACAAAATGGCAGCCATAAAGCCAGA |
| ONG-Ssndt80b-R | CATCTTATCTACATACGCTACTATGCGGCGCTCCAAGTGTAGT |
| ONG-Ssndt80c-F | GAATGGATGAACTTTACAAAATGGCAGCTTATGATACTCT |
| ONG-Ssndt80c-R | CATCTTATCTACATACGCTATCAATCGTCTGCTGAGAAGTATC |
| ONG-SsSNF1-F | GAATGGATGAACTTTACAAA ATGAATCGTTATGATGACGG |
| ONG-SsSNF1-R | CATCTTATCTACATACGCTA TTAATCAGCTTCTGCCAATT |
| 1301GFP-Ssndt80b-F | AGAACACGGGGGACGAGCTCATGGCAGCCATAAAGCCAGA |
| 1301GFP-Ssndt80b-R | CCGCTACCGTCGACTCTAGATGCGGCGCTCCAAGTGTAGT |
| LUC-CREAmotif-F | TTCCTGCAGCCCGGGGGATCTTTCTCTCCAATGATTTGAT |
| LUC-CREAmotif-R | CGGCCGCTCTAGAACTAGTGTAAAGTGGGTTATGTTCACT |
| LUC-SNF1motif-F | TTCCTGCAGCCCGGGGGATCCGGATGGAATCAAAAATCTG |
| LUC-SNF1motif-R | CGGCCGCTCTAGAACTAGTGGGCGTTGAGTTGTGAAGTAT |
| AD-Ssndt80a-F | ATATGGCCATGGAGGCCAGTATGGGGATGCGATATTTGAT |
| AD-Ssndt80a-R | ATCTGCAGCTCGAGCTCGATCTAGCTGATATTAAGCTCCT |
| AD-Ssndt80b-F | ATATGGCCATGGAGGCCAGTATGGCAGCCATAAAGCCAGA |
| AD-Ssndt80b-R | ATCTGCAGCTCGAGCTCGATCTATGCGGCGCTCCAAGTGT |
| AD-Ssndt80c-F | ATATGGCCATGGAGGCCAGTATGGCAGCTTATGATACTCT |
| AD-Ssndt80c-R | ATCTGCAGCTCGAGCTCGATTCAATCGTCTGCTGAGAAGT |
| pabai-SsSNF1-F | AAAAAAAATGATGAATTGAACGGATGGAATCAAAAATCTG |
| pabai-SsSNF1-R | TCGAGGTCGACAGATCCCCGGGCGTTGAGTTGTGAAGTAT |
| pabai-SsCREA-1-F | AAAAAAAATGATGAATTGAAGACGGATGGGGGAAGGAAGG |
| pabai-SsCREA-1-R | TCGAGGTCGACAGATCCCCGAAATACGCTAAACTAGTGTC |
| pabai-SsCREA-2-F | AAAAAAAATGATGAATTGAAGCAAAAAAGAAATCATACCA |
| pabai-SsCREA-2-R | TCGAGGTCGACAGATCCCCGTCAAAAATGCCGAAAACTTT |
| pCold-Ssndt80b-F | GGCATATGGAGCTCGGTACCATGGCAGCCATAAAGCCAGA |
| pCold-Ssndt80b-R | GACTGCAGGTCGACAAGCTTCTATGCGGCGCTCCAAGTGT |
| P-SsSNF1-F-BIOTIN | TGAAGAGCAGCTGGAACCACAAATCAACTTTTCCTTTAA |
| P-SsSNF1-R | TTTAAAGGAAAAGTTGATTTGTGGTTCCAGCTGCTCTTCA |
| P-SsSNF1-F | TGAAGAGCAGCTGGAACCACAAATCAACTTTTCCTTTAAA |
| MP-SsSNF1-F | TGAAGAGCAGCTGGAACAAAAAATCAACTTTTCCTTTAAA |
| MP-SsSNF1-R | TTTAAAGGAAAAGTTGAAAAAAAGTTCCAGCTGCTCTTCA |
| Q-SsActin-F | GAATGTGTAAGGCCGGTTTCGC |
| Q-SsActin-R | CATCCCAGTTGGTGACGACACC |
| Q-Ssndt80a-F | CTCGCACTCCAGGCGATATT |
| Q-Ssndt80a-R | GAGTGGGCTCCATGGGAATC |
| Q-Ssndt80b-F | GCGAACAATGGTCGAAGACG |
| Q-Ssndt80b-R | TTCATGGTAGCACTGGCAGG |
| Q-Ssndt80c-F | ACTGCTAGACCTACGACCCA |
| Q-Ssndt80c-R | ATCGCATCTACTGGTGGCAT |
| Q-SsPKS12-F | TCCACGACGCATCTTTGTCA |
| Q-SsPKS12-R | ATGCTGTATCCTACGTGCCG |
| Q-SsTHR1-F | GAAATCGAGGGGCTCGGTAG |
| Q-SsTHR1-R | ACCGGACATATTGGCAGCAA |
| Q-SsSCD1-F | ACTCGAATCCATCATCGCCC |
| Q-SsSCD1-R | TCGCATGACTGTGTCCCTTC |
| Q-SsPKS13-F | CGCTGATGGTTATTGCCGTG |
| Q-SsPKS13-R | TGCGATACCAGCGATGACTC |
| Q-SsCREA-F | CCGCTATTGGTAGCCCGAAT |
| Q-SsCREA-R | GTCTGTGAGAGGGGCTTGAC |
| Q-SsSNF1-F | GGCTTTGGCTGCAGAAGAAC |
| Q-SsSNF1-R | GATACGGCATCGAGAAGGGG |
| Q-SS1G_07749-F | TCAGCGGAACCTTCGGAATC |
| Q-SS1G_07749-R | AACTGCCTTTTACTGTGCCGA |
| Q-SS1G_10092-F | GCTGGACTGGAAACAAGGGA |
| Q-SS1G_10092-R | GTAGACTGAGCCGTCGGATG |
| ChipQ-SsTubP-F | TCACGTGATTGAGAATGTTC |
| ChipQ-SsTubP-R | TGAGATGTCCTTGTGGATAG |
| ChipQ-SsSNF1-F | CCTAGAGCTTCACGCATCGG |
| ChipQ-SsSNF1-R | TCTTCCGTGGAGAATTAAAC |
| ChipQ-SsCREA-1-F | CTCCAATGATTTGATTAGAG |
| ChipQ-SsCREA-1-R | AAGAGGCCAGTTATCTATAC |
| ChipQ-SsCREA-2-F | AATTCAAAAAATCCCACACT |
| ChipQ-SsCREA-2-R | TTAGTACGTATAGTAACTAG |
| ChipQ-SsCREA-3-F | ATACATCCCCCCATCCCGTA |
| ChipQSsCREA-3-R | GGCCGAAGCGGAGGAGAGCG |
